# Facing the “New Normal”: How adjusting to the easing of COVID-19 lockdown restrictions exposes mental health inequalities

**DOI:** 10.1016/j.jpsychires.2021.07.001

**Published:** 2021-07-06

**Authors:** Naomi A. Fineberg, Luca Pellegrini, David Wellsted, Natalie Hall, Ornella Corazza, Valentina Giorgetti, Dorotea Cicconcelli, Elena Theofanous, Nick Sireau, David Adam, Samuel R. Chamberlain, Keith R. Laws

**Affiliations:** 1 School of Life and Medical Sciences, University of Hertfordshire, Hatfield, United Kingdom; 2 Hertfordshire Partnership University NHS Foundation Trust, Welwyn Garden City, United Kingdom; 3 University of Cambridge School of Clinical Medicine, Cambridge, United Kingdom; 4 Department of Biomedical and Neuromotor Sciences, University of Bologna, Italy; 5 University of Southampton, Department of Psychiatry, Faculty of Medicine, Southampton, United Kingdom; and Southern Health NHS Foundation Trust, Southampton, United Kingdom

## 1 Background

In March 2020, the UK Government enforced its first national ‘lockdown’ in response to the COVID-19 pandemic. Significant restrictions to the lifestyle and conduct of the public were enforced, including institution of various safety-behaviours such as washing, mask-wearing and physical distancing, aimed at reducing the spread of infection. From July – November 2020, as infection rates dropped, a gradual easing of restrictions occurred in most parts of the country alongside a short-lived restoration of more normal ways of living. In other areas where rates remained high, a partial lockdown was re-enforced, sometimes at very short notice. Similar unpredictable changes in levels of restriction and control continued to be applied over subsequent months and, as a new viral strain emerged in the UK around December 2020, another full lockdown was implemented at a national level.

During the brief period of partial “lockdown-release” in Summer 2020, the public was incentivized to return to school, work, universities, shops and restaurants e.g. via the ‘Eat Out To Help Out’ scheme (The Guardian, 2020), driven by a number of motivations, including the need to reinstate vital public services such as health and education and to support the UK economy, which has continued to suffer (The Guardian, 2020). Re-establishing social activity was also considered important for public mental health and psychosocial wellbeing (World Health Organization, 2021), in particular, the mental health and psychosocial development of children and young people (Lewis et al., 2021).

The success of such initiatives would however depend on individuals flexibly adapting their behaviour to the changed conditions, in the face of residual uncertainty about personal risk and the risk they posed to others. In Summer 2020, many people still expressed anxiety about leaving their homes for fear of catching or transmitting the virus. Those in demographic groups considered to be vulnerable to infection (elderly, black and minority ethnicities, immuno-compromised, physically frail), or working in higher risk environments (frontline health and care services, schools), expressed particular apprehension (Egede et al., 2020). Feedback received during the study suggested at least some members of the public found it easier adjusting to the introduction of the rules at the beginning of lockdown than to their easing because of difficulties managing contradictory information and advice.

Very little is known from this or other recent coronavirus pandemics (e.g. severe acute respiratory syndrome) about the ways in which the public responds to the easing of pandemic restrictions and the impact of mental disorder on this response (Savage, 2020; Peng et al., 2010). As problems adjusting ‘post-pandemic’ are likely to impact longer-term wellbeing, societal functioning and prosperity at both the individual and public health level, such information would be of relevance for guiding public-health and clinical healthcare policy in the interests of improved long-term public-health in the aftermath of the pandemic.

Based on clinical evidence, those with obsessive-compulsive (OC) and related disorders (OCRDs), symptoms or personality traits (e.g. cautious, rule-bound, habitual, rigid, conscientious) (APA, 2013), representing approximately 20% general population (Fineberg et al., 2013), would be expected to find adjustment difficult (Fineberg et al., 2015; Fineberg et al., 2018; Apergis-Schoute et al., 2017). Therefore, we consulted with individuals with lived experience of obsessive-compulsive disorder (OCD) (patient and public involvement representatives), with whom we designed and ran the study. Individuals with OC symptoms are known, from laboratory-based testing, to have particular difficulty selecting adaptive behavioural responses under conditions of uncertainty (Morein-Zamir et al., 2020; Vaghi et al., 2017). They also demonstrate a tendency to behave habitually under conditions of threat and show difficulty flexibly ‘unlearning’ previously rational responses to danger once the danger has passed (Gillan et al., 2015). Some preliminary studies have demonstrated a significant worsening of symptomatology, including exaggerated precautionary behaviours and difficulty managing uncertainty, among treatment-seeking patients with OCD during the early stages of the outbreak (Benatti et al., 2020; Van Ameringen, 2020), but this finding is not consistent in all of the studies (Zohar, 2020; Jelinek et al., 2021). Thus, individuals with OCD (Fineberg et al., 2020; The Guardian, 2020) may be expected to find relinquishing behaviours previously endorsed as being necessary for protection against infection from COVID-19 particularly challenging.

A few studies during the pandemic have employed online sampling to examine OC symptoms (or tendencies) and their impact in general population samples; however, none so far have examined their effect on post-pandemic adjustment. A cross sectional survey conducted in Turkey early in the pandemic (Seçer & Ulaş, 2020) found that fear of Covid-19 acted as a predictor of OC symptoms in the general population. Another large cross-sectional survey of OCD at the peak of the first wave, conducted in Canada in people with chronic mental or physical illnesses, healthcare providers, and the general population (Robillard et al., 2020), found that in the 4920 of 6040 participants who provided OCD scores, higher scores on the contamination subscale were significantly related to increased self-reported stress. A study by Samuels et al. (2021) recruited 2117 US-based individuals and found increases in OC symptoms, and in particular contamination obsessions, during the pandemic, which were significantly correlated with Covid-19 related safety behaviours. Interestingly, these relationships remained significant whether or not a person reported a prior diagnosis of OCD. In a sample of 829 US-based adults, Fontenelle et al. (2021) found that OC and related symptoms had significantly worsened during the pandemic and this was predicted by female gender, stressful pandemic-related life-events and higher compulsivity. Similarly, Albertella and colleagues (2021) found that self-reported OCD symptoms significantly increased from before to during the pandemic in an online sample of 992 adults aged 18-84 years. Furthermore, OCD symptomatology during lockdown was positively associated with Covid-related stressful life events and compulsivity traits measured on the Cambridge-Chicago Compulsivity Trait Scale, as well as younger age and psychological distress. Another US survey by Wheaton et al. (2021), reported a positive correlation between fear of Covid-19 spread and other measures including OCD symptoms. Regression modelling suggested that intolerance of uncertainty partly statistically accounted for the link between OC symptoms and Covid-19 concerns.

There has also been a small number of longitudinal population-based.studies investigating OC symptoms. Meda et al. (2021) investigated 358 Italian university students and reported that OC symptoms did not significantly differ before and during the pandemic, but reduced significantly when lockdown was lifted. In contrast, Knowles and Olatunji (2021) investigated 128 US university students and found washing symptoms of OCD significantly increased after the onset of the pandemic, while a study of >4000 Chinese university students (Ji et al. 2020) showed significant improvement in OC symptoms as the pandemic eased.

Therefore, emerging evidence suggests that OC symptoms, in particular those related to contamination fears, have increased during the pandemic in general population samples, may have declined as the pandemic eased, and are associated with pandemic-related stress and/or fear (Robillard et al., 2020, Seçer and Ulaş 2020, Albertella et al., 2021, Fontenelle et al., 2021), as well as with trait compulsivity (Albertella et al., 2021, Fontenelle et al., 2021).

Clinical studies have additionally shown evidence of latent cognitive inflexibility among those with OCD or other OCRDs (e.g. body dysmorphic disorder, hoarding disorder); extending also to those with obsessive compulsive personality disorder (OCPD) – a syndrome characterised by the need for perfection, completeness and certainty, extreme conscientiousness and stubbornness (APA, 2013). Studies have documented this inflexibility on the intra-dimensional extra-dimensional (ID-ED) set-shift task from the Cambridge Neuropsychological Test Automated Battery (CANTAB) (https://www.cambridgecognition.com), a modified form of the Wisconsin Card Sort Test, which probes components of rule-acquisition and reversal learning capabilities, requiring maintenance, shifting and flexibility of attention and which is sensitive to rigid response-tendencies (Fineberg et al., 2015; Chamberlain et al., 2021; Chamberlain et al., 2005). The online version of the ID-ED has been validated in patients and community-based samples (Sternin et al., 2019), but its use has not so far been reported in the evaluation of post-pandemic adjustment.

## 2 Aims and Objectives

This study aimed to identify the extent to which difficulties adjusting to the easing of lockdown conditions experienced by the public relates to existing mental health problems, and the specific demographic or health-related factors mediating these associations.

We hypothesised that those with a personal history or family history of mental disorders, and those expressing OC symptoms or traits, would find adjustment most difficult; and that those experiencing greater adjustment problems would show increased levels of cognitive inflexibility on an objective online cognitive test of set-shifting (ID-ED: Robbins et al., 1998).

## 3 Methods

The protocol and study objectives were pre-registered on 15th July 2020 (Open Science Framework; doi:10.17605/OSF.IO/GS8J2). Ethics approval was granted from the University of Hertfordshire Health, Science, Engineering and Technology Ethics Committee with Delegated Authority (Ethics number: aLMS/SF/UH/04219). The study ran from 16/07/2020 to 13/10/2020, during which period pandemic restrictions were partially eased in the UK - with schools, universities and high street shops re-opened and people were allowed to travel and mix socially, albeit with some limitations.

### 3.1 Participants

Participants were recruited from a broad spectrum of the general adult population. Although UK-residents aged 18+ years were targeted and the questionnaires were in English, no geographical or age restrictions were applied. A ‘snowball sampling’ technique (Encyclopedia of Survey Research Methods, 2008) was employed; with the study and its objectives being advertised on the radio, social media platforms, including Facebook groups (e.g., University groups and Fitness groups), LinkedIn, WhatsApp, Twitter, and Instagram and also on OCD-related website/groups. We targeted recruitment of those living with anxiety and OCD, as we wished to ensure adequate representation of people with pre-existing mental disorder including OCRDs, who are known to respond less frequently to surveys (Pierce et al., 2020).

### 3.2 Design

This is a cross-sectional study consisting of a two-phase web-based survey, hosted on Qualtrics Software (Snow & Mann, 2008). The first phase investigated adjustment alongside demographic and clinical variables. All participants gave written informed consent and were asked if they would also consent to be contacted for phase 2. The second phase involved an online assessment of cognitive flexibility in two subgroups designated as either poor-adjusters or good-adjusters.

Phase-2 participants were recruited on a ‘first-come first-served basis’ from UK respondents who consented in phase 1; and were grouped according to their response to the first of a series of questions evaluating ability to adjust (described below and in Table 1). Designated poor-adjusters were consecutively contacted by email and sent a digital link to complete the cognitive task, until a total of 20 had been returned. Each poor-adjuster recruited was matched with a good-adjuster according to age, gender and educational level. At analysis, we excluded any results confounded by missing data or distraction (as defined automatically by the CANTAB online task when participants were distracted by external stimuli).

### 3.3 Outcomes

#### Phase one

We first gathered demographic and clinical details including history of having contracted COVID-19 or a family member having done so, history of bereavement through COVID-related illness, personal history or family history of mental disorders (including OC and related disorders). We also asked a question to identify the extent to which the individuals judged they had complied with Government guidance during the lockdown period.

Clinical variables were examined using the following self-rated questionnaires previously validated in population-based studies (Henry et al., 2005; Fineberg et al., 2015; Chandu et al., 2020; Fiorillo et al., 2020).

- Depression, Anxiety and Stress Scale (DASS-21) (Lovibond & Lovibond, 1995): A self-assessment scale measuring the severity of anxiety (7 items), stress (7 items) and depressive (7 items) symptoms.
- Obsessive-Compulsive Inventory-Revised (OCI-R) (Foa et al., 2002): An 18-item self-rated scale for assessing obsessive-compulsive symptom severity. A score of >=21 points indicates the likely presence of OCD.
- Compulsive Personality Assessment Scale (CPAS) (Fineberg et al., 2015): An 8-item self-rated (or observer-rated) instrument measuring the severity of individual traits of DSM-5 OCPD. The CPAS has been found to differentiate individuals with OCPD both in a university student sample (Fineberg et al., 2015), where it was validated against an objective measure of cognitive inflexibility (ID-ED task), and among various clinical groups of patients.
- COVID-19 Anxiety Scale (18) (CAS) (Chandu et al., 2020), a 7-item self-rated scale measuring the extent of anxiety related to COVID-19 infection.

Finally, the extent to which participants experienced adjustment difficulties following the release of lockdown was assessed using seven likert-type statements (see Table 1).

#### Phase two

Poor adjusters were identified as those who *agreed* or *completely agreed* with the statement “I am having great difficulty adjusting to the easing of the Covid-19 pandemic restrictions”, while good adjusters were identified as those who *disagreed* or *completely disagreed* with the same item. From among those who agreed to participate in Phase 2 and met either of these criteria (see Design), we sent a link to an online version of the ID-ED task. As this was an exploratory analysis, the estimated sample size (20 per group) was based on previous studies using the ID-ED task with comparable groups (Fineberg et al., 2015). Alongside overall performance on the task, representing a global measure of cognitive inflexibility and indicated by the number of trials completed on all attempted stages, we were interested in performance on the specific items evaluating extradimensional set-shifting and reversal (respectively stages 8, 9), which in previous studies of individuals with OC symptomatology or traits have demonstrated sensitivity for capturing cognitive inflexibility (Fineberg et al., 2015; Fineberg et al., 2018; Chamberlain et al., 2005).

### 3.4 Statistical Analyses

First, the descriptive information and correlation matrix were examined. Shapiro-Wilks test was used to detect any departure from normality. Second, we followed the 4-step procedure outlined by Baron & Kenny (1986) to establish mediation effects. All analyses were conducted in JASP 0.13.1. We tested a serial mediation model with OCI-R, CPAS and mental health history as predictors, DASS-21 and Covid anxiety as mediators and adjustment as outcome. The first step was to establish that our initial and pre-registered predictor variables (OCI-R, CPAS) were correlated with the outcome (Adjustment). The second step involved showing that the initial predictor variables also correlated with our mediators (Covid anxiety and DASS-21 scores). The third step established the associations between our mediators (Covid anxiety and DASS-21) and the outcome (Adjustment). In this latter step, the assumption is that the correlation between the mediator and the outcome variables exists because both are related the initial variable. Finally, we established full mediation across both mediators. An exploratory analysis of ID-ED total scores and stages comparing good versus poor adjusters that took part in the neurocognitive task was performed using Mann-Whitney U-tests. Statistical significance was set at p=.05 across all tests.

## 4 Results

### 4.1 Participants

**Figure 1:**
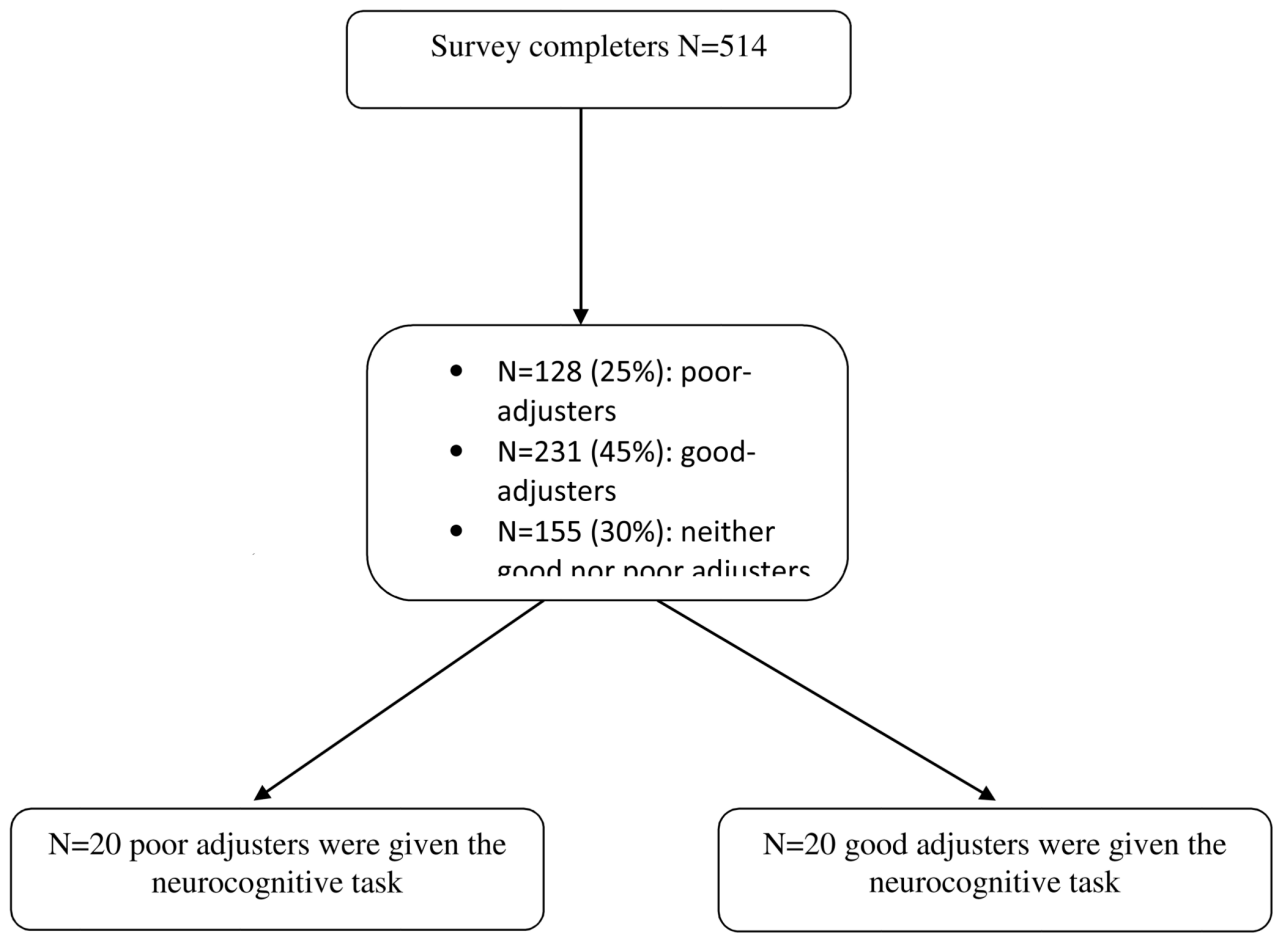
Flow-chart of participants.

Phase 1 was completed by 514 participants (Figure 1). Table 2 lists their demographics. As per similar surveys (Smith, 2008), the majority of respondents were female and most were well-educated. The mean age of the sample was 36.7 (SD=13.3) years. They mainly derived from the UK (90%); 6% from Italy and 4% from other European countries or the USA. All of these countries underwent a similar temporary release from lockdown over the study period (BBC, 2020). Of the sample, 85% self-identified as of white ethnic status and 65% were employed, with a roughly equivalent minority either unemployed (10%) or furloughed (8%).

Table 4 lists the clinical characteristics of the sample. The mean DASS-21 (32.12, SD: 15.73) and its subscales were numerically higher than normative pre-COVID scores^29^ but similar to other general population findings reported during COVID-19 (Burke et al., 2020; Fiorillo et al., 2020), and in particular during March-April-May 2020 (Fiorillo et al., 2020), suggesting that the depressive/anxious/stress symptoms may have carried over after release from lockdown. In line with previous studies conducted during lockdown (Fiorillo et al., 2020), the stress subscale of DASS-21 was the most elevated (mean 12.08, SD:5.79), implying a relatively greater contribution from stress (see Table 4).

The mean OCI-R score was also elevated at 32.5 (SD = 11.7), with 31% of those reporting a history of mental illness and 21% of those with no previous history of mental illness scoring higher than the screening threshold for OCD (OCI-R >=21) (Foa et al., 2002). This finding suggests a substantial incidence of new cases of OCD having developed during the pandemic, although without a clinical assessment this cannot be confirmed. The sample also showed a mean CPAS score of 16 (SD=8.4), which was compatible with the mean score of 17 seen in a previous study in a population-based sample diagnosed with OCPD (Fineberg et al., 2015). A large proportion of respondents (85%) said they closely followed the safety rules.

### 4.2 Adjustment across the whole sample

The sum of scores on the 7 adjustment statements correlated significantly with all the clinical scales (see Table 5).

A significant positive correlation (small) was found between adherence to COVID-related rules and OCI-R (r = .15, p=.002) and a significant moderate negative correlation (r = .27; p<.001) between adherence to the same rules and adjustment (total score).

### 4.3 Mediation analysis

#### A Dimensional Analysis (total sample)

We conducted a mediation analysis for the whole sample using JASP 0.13.1. The total score across the 7 adjustment statements was used as the outcome. The predictors and mediators of adjustment were chosen based on our pre-registered a-priori hypotheses: Given our a-priori hypothesis that OC symptoms (OCI-R) and traits (CPAS) along with previous mental health history could be risk factors, these were entered as predictors of adjustment, while DASS-21 and CAS scores were entered as mediators.

In this model, none of the predictor variables had a direct effect on adjustment. Nevertheless, all predictors (history of previous mental disorder, OCI-R and CPAS) had significant indirect impacts on adjustment, via the two predetermined mediators. Previous history of mental disorder significantly predicted adjustment problems acting through the DASS-21 (z-score: 3.03, p=0.002), whereas the OCI-R score was a significant predictor of adjustment via both the DASS-21 (z-score: 3.22, p=0.001) and the CAS (z-score: 7.37, p=.001). The CPAS score was also significantly related to total adjustment scores via the DASS-21 (z-score: 2.82, p=0.005). The model accounted for 53% of the variance in the adjustment outcome measure.

#### B Categorical Analysis (poor versus good adjuster subgroups)

One hundred and twenty-eight (25%) participants were classified as poor-adjusters, based on the *a priori* definition relating to the first adjustment question (Table 1); whereas 231 (45%) were classified as good-adjusters. One hundred and fifty-five (30%) endorsed ‘neither agree nor disagree’ and were designated ‘indeterminate-responders’ and were excluded from the analyses reported below.

The good and poor adjustment groups did not differ in: age (t=1.79, p=0.18), sex (χ^2^=2.81, p=0.25) or level of education (χ^2^=2.27, p=0.99). Compared to good-adjusters, the poor-adjusters reported a higher incidence of history of mental disorder, both personal (χ^2^=8.61, p=0.003) and in their family (χ^2^=7.52, p=0.04). Poor-adjusters also reported significantly higher COVID-related anxiety on the CAS (t=5.64, p< .001), more depressive/anxious/stress symptoms on the DASS-21 (t=3.89, p=< .001), more OC personality traits on the CPAS (t=4.55, p=< .001) and more OC symptoms on the OCI-R (t=2.93, p=0.004).

We ran a mediation analysis using JASP 0.13.1 using the predictors and mediators outlined previously, but with a categorical outcome of poor versus good adjustment. In this model, previous history of mental disorder directly impacted adjustment (z-value: 2.64, p=0.008). OCI-R score was also a significant predictor of adjustment, but indirectly via CAS scores (z-value: 5.28, p=0.001). No other effects were significant.

### 4.5 Neurocognitive data

Fifty-five percent of our sample (N=282) consented to participate in phase 2. Of these, 20 consecutive responses from the poor-adjusters and 20 from a demographically-matched subgroup of good-adjusters were analyzed (see Design). Five datasets (2 from poor-adjusters, 3 from good adjusters) had to be excluded from the final analysis owing to missing data or evidence of distraction during task performance.

Good and poor-adjuster subgroups did not differ in age, sex or educational level. The ID-ED Total Trials score significantly differed between good and poor-adjusters, the latter group performing worse (Cohen’s d 0.41, Mann-Whitney U test, p: 0.03), as did the Total Trials on stage 9 score (extradimensional shift reversal) (Cohen’s d: 0.79, Mann-Whitney U test, p: 0.02). No significant between-group difference was found on the Total Trials on stage 8 score (extradimensional set-shift) though poor-adjusters showed a numerically poorer performance on this item also (Cohen’s d: 0.17, p: 0.2).

## 5 Discussion

Much research has focused on how the COVID-19 lockdown itself impacts mental health and well-being and some research has addressed the impact of lockdown on the health of those living with mental disorders (Nemani et al., 2021). By contrast, research into how we adjust to the release from lockdown has been overlooked. As far as we are aware, the current study is the first to investigate the factors that might impact adjustment following the release of COVID-19 lockdown restrictions.

**Figure 2:**
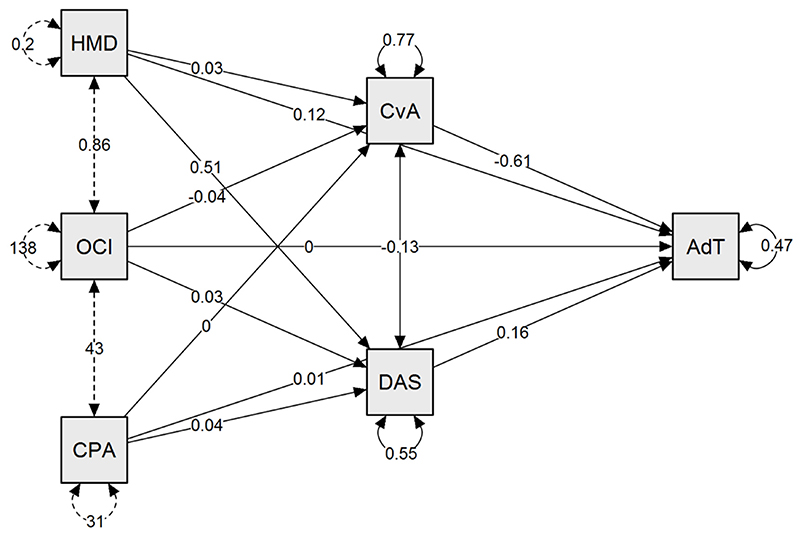
Mediation model of the total sample This path plot is a graphical representation of the mediation model for the total sample (Table 6). The mediation is showing that the effect of designated variables -history of mental disorder (HMD), OC symptoms (OCI-R) and OC traits (CPAS) - on adjustment is indirect, acting via other variables: fear of COVID (CAS) and depressive-anxious-stress symptoms (DASS-21). History of mental disorder, OC symptoms (OCI-R) and OC traits (CPAS) are the predictors; fear of Covid (CAS) and depressive-anxious-stress symptoms (DASS-21) are the mediators; adjustment is the outcome. In the analysis, adjustment is calculated as the total sum of the 7 bespoke questions (score 1 to 5 for each question). The arrows indicate the effects, that can be direct or indirect. The total effect is the sum of the direct and the indirect effect: mediation analysis decomposes an existing effect into these two terms. In the path plot the standardized values of the relationship (strength of the paths) are reported on the links. AdT: Adjustment CvA: Covid Anxiety Scale (fear of Covid) DAS: DASS-21 HMD: History of mental disorder CPA: CPAS OCI: OCI-R

Our sample of over 500 individuals included a fair representation of those with mental health disorders of relevance to our research question. A central finding was that one-in-four of our sample reported struggling to readjust following lockdown release and that those with pre-existing mental disorders were disproportionately affected. Indeed, when evaluating all respondents, a history of mental disorder and the presence of OC symptoms (measured on the OCI-R) and personality traits (measured on the CPAS) had an indirect impact on adjustment via depression, anxiety and stress (DASS-21), of which stress was the major mediating factor; while OC symptoms impacted adjustment through COVID-related anxiety (CAS). When comparing those identified as at the extremes of poor and good adjustment, having a previous history of mental disorder had a *direct* statistical effect on adjustment (Figure 2). In this model, the presence of OC symptoms significantly affected adjustment, but indirectly via increased COVID-related anxiety, and not via changes in depression, general anxiety or stress.

Individuals with a history of any mental disorder therefore appear to be disproportionately affected and may struggle to adjust to the lifting of lockdown. Individuals in the general population with OCD symptoms (obsessions, compulsions) and those expressing OC personality traits (e.g. rigid, perfectionist, conscientious, detail-focused) also found adjusting difficult. OC symptoms, which are common, affecting up to 20% of the general population (Fineberg et al., 2013), acted on adjustment largely by inducing stress and anxiety as well as more specific fear of COVID-19 infection. By contrast, perfectionist or rigid personality traits, which are also common (Fineberg et al., 2015) and may be adaptive in certain situations (Hertler et al., 2015), adversely affected the adjustment process mainly by increasing stress and anxiety.

Our survey-based results are consistent with findings from clinical research showing the occurrence of heightened symptomatology, distress and functional disability in some patients with OCD during the outbreak of the pandemic (Benatti et al., 2020). Worsening was noted in a prior study (Jelinek et al., 2021), especially among those with pre-existing obsessions about contamination, for whom the fear of contracting or spreading COVID-19 exacerbated compulsive washing, cleaning, checking and avoidance of going out. In this study (Jelinek et al., 2021), OCD patients with washing compulsions also showed greater confidence in providing other people with advice related to infection- prevention. Patients with OCD have even expressed doubts about the rationality of the evidence-based therapies aimed at reducing compulsive activities that they had been pursuing (Varinelli et al., 2021). The findings that adherence to COVID-related rules correlated positively with OC-symptoms and negatively with adjustment, while unable to indicate causation, nevertheless raise the possibility that those with OC symptoms who showed strict adherence to mandated washing or social-avoidance during the pandemic may be at increased risk of adjustment problems when the pandemic ends.

The current study focused on how existing mental disorders, as well as OC symptoms or OC personality traits might impact adjustment in the general population. We advertised the study’s objectives, which may possibly have inflated the proportion of respondents experiencing adjustment problems (Perlis et al., 2021). We also attempted to ensure participants with a history of mental disorders including OCD were not under-represented. However, most of the sample did not have adjustment difficulties and the overall rate of reported mental disorder was 30%. This latter rate is comparable to the clinically significant rates of mental distress (27.3%) reported in the UK population in 2020 during the COVID-19 pandemic (Pierce et al., 2020) as contrasted to rates of mental disorder usually found in the general population (around 20%) (McManus et al., 2016). The rates of OC symptomatology in our sample were, however, relatively high compared to population norms (Varinelli et al., 2021), with 31% scoring above the screening-threshold for possible OCD (perhaps reflecting our recruitment strategy). Nevertheless, one fifth of our sample (21%) with no previous mental disorder scored 21+ on the OCI-R, suggesting a possible effect of the pandemic on the reporting of OC symptoms. It remains to be seen whether the pandemic will have a lasting effect on rates of mental disorders including OCD. Our study was unable to explore the extent to which OC symptoms among the general population may have diminished once the pandemic eased, as reported by Meda et al (2021) and Ji et al (2020). Our own clinical experience shows evidence of cases with enduring symptoms of OCD that first arose during the pandemic, precipitated by various complex factors. For example, a teenager developed severe OCD after being advised to change her clothes after returning from school to safeguard her mother’s health. On this point, we note that only a minority of our sample reported lived experience of COVID-19 infection (14% answered yes to the question: “Have you or any member of your family contracted COVID-19?”). Therefore, we might infer that our results are not related to a physiological post-viral inflammatory syndrome sometimes described as “long-COVID” (Mandal et al., 2020), but to the psychological effects of living through the pandemic.

We acknowledge that it may be possible that OC symptoms during the pandemic are to some extent adaptive. However, while such symptoms may promote adherence to regulations that increase safety under unusually risky conditions, such as pertained under this pandemic, once the risk has passed and normal risks are restored, they no longer confer this advantage - and according to our a priori hypothesis – may even interfere with normal adjustment processes. Our study investigated the longer-term consequences of OC symptoms and, in line with our hypotheses, found they predicted adjustment problems in the aftermath of the pandemic.

Our findings have implications for public health and clinical services. Further research will be required to determine the clinical interventions and services of most value to aid adjustment in those with a history of mental disorders or OC symptoms and personality traits. We might expect that evidence-based psychosocial strategies currently used to support and improve functional activity for patients with anxiety, stress and depression, such as different forms of cognitive behaviour therapy (CBT), or possibly evidence-based pharmacotherapy such as use of a selective serotonin reuptake inhibitor in cases where symptoms of anxiety and depression are more severe, would be helpful. For those already in receipt of mental healthcare, adaptations to the roles of occupational therapists, CBT therapists and support workers, either working alongside general practitioners in primary mental healthcare services such as Improving Access to Psychological Therapies Services (https://www.england.nhs.uk/mental-health/adults/iapt/) or in designated community mental health teams may facilitate this function.

Cognitive inflexibility was apparent amongst poor-adjusters on key domains of the ID-ED task. This represents an objective measure of cognitive inflexibility known to discriminate those with various OCRDs from healthy controls and other clinical groups (Fineberg et al., 2018), thereby increasing confidence in the subjective clinical ratings (OCI-R, CPAS) that correlated with poor adjustment. Poor-adjusters performed most poorly on the reversal learning aspect of the ID-ED shift task, which indicates the inability to relinquish a behavior when it is no longer appropriate, and hints that individuals with this performance deficit might have greater difficulty relinquishing safety-behaviours, opening up new research avenues. Impaired reversal learning specifically at this stage of the ID-ED task has been correlated with restricted interest repetitive behaviour symptoms in a study of patients with autism spectrum disorders (Yerys et al., 2009). Intriguingly, in that study also, extradimensional set-shift (stage 8) was only numerically impaired to a non-significant degree.

Furthermore, as cognitive inflexibility on the ID-ED task reflects a latent phenotype, our findings suggest that OC symptoms or traits may influence post-pandemic adjustment partially via impairment in executive function. This has further implications for therapeutic intervention, as CBT seems to be less effective in those with this form of executive dysfunction (D’Alcante et al., 2012). Moreover, ‘cognitive remediation’ techniques tackling cognitive inflexibility in those with compulsive disorders have not so far emerged as reliably effective in randomized trials (Van Passel et al., 2020). New research heuristics may then be required to develop effective interventions. Scoping work identifying possible treatment options for the cognitive-functional difficulties associated with OCD, such as activity scheduling and habit-reversal therapy (HRT) (Varinelli et al., 2021), may act as a rational starting point.

### 5.1 Strengths and Limitations

This study had a number of strengths, including preregistration, the use of standardized self-rating scales and objective neurocognitive testing. Although a small minority of participants resided outside the UK, all participants were subject to broadly the same conditions of release during the study period. Nonetheless, several limitations should be considered. First, the cross-sectional nature of the study means that while we can determine statistical mediation, we cannot confirm the causal nature of the identified associations. By following-up participants with an objective test of cognitive flexibility (ID-ED), we addressed some of the limitations of our subjective survey design. However, owing to the preliminary nature of this research, our sample in phase 2 was inevitably small and the analysis focused exclusively on the extremes of adjustment. Replication in a larger sample including indeterminate responders is therefore recommended to better understand the relationship between cognitive inflexibility and adjustment.

## 6 Limitations

Our study is subject to a number of limitations. First, there is a potential risk of sampling bias; for example, those responding to the survey could have been those experiencing relatively more anxiety, stress, depression or difficulties in adjusting to the release of restrictions. However, we do not consider this would have undermined our major objectives, which were to identify those experiencing adjustment difficulties and the factors that might mediate such difficulties. Secondly, we must acknowledge that there may have been minor inconsistencies in the levels of restrictions our participants were subjected to. However, as more than 90% of the participants were sampled from across the UK (87% from England and 4% from Scotland), and most UK regions were subjected to similar levels of restriction (and release) over the study period (July-October 2021), and a further 6% of participants were from Italy (see Table 2) and therefore undergoing a similar phase of release from restrictions as the UK (BBC, 2020), we believe that the level of inconsistency is too small to have substantially affected our results. Thirdly, we did not differentiate adjustment between those who had continued to work during the lockdown and those who were working from home; and so, we do not know if continuing to attend the workplace afforded better adjustment to lockdown release. Fourthly, we defined adjustment exclusively in terms of subjective difficulties experienced by the individual. It would have been informative to additionally assess other forms of adjustment, such as occupational or social functioning (for instance, days not working, sick leave etc.). Future research should incorporate a broader functional assessment to provide more information on the impact and cost of the adjustment difficulties. We additionally acknowledge that our set of bespoke items assessing adjustment has not been externally validated; however, we could not locate any existing validated measure for this purpose.

## 7 Conclusions

While it might be assumed that release from lockdown is a universally positive experience, our data indicate that as many as one-in four people experience adjustment difficulties and this is a particular problem for those with a history of mental disorder. We also report that individuals with OC symptoms or rigid, perfectionist personality traits may be especially vulnerable to adjustment problems, even when they have no history of a formal mental health diagnosis. This paper highlights the risks that those with existing mental health problems may be further disadvantaged – crucially, when the pandemic ends, if this is not proactively addressed through the development of new clinical and public health policies and interventions.

## Figures and Tables

**Figure 3:**
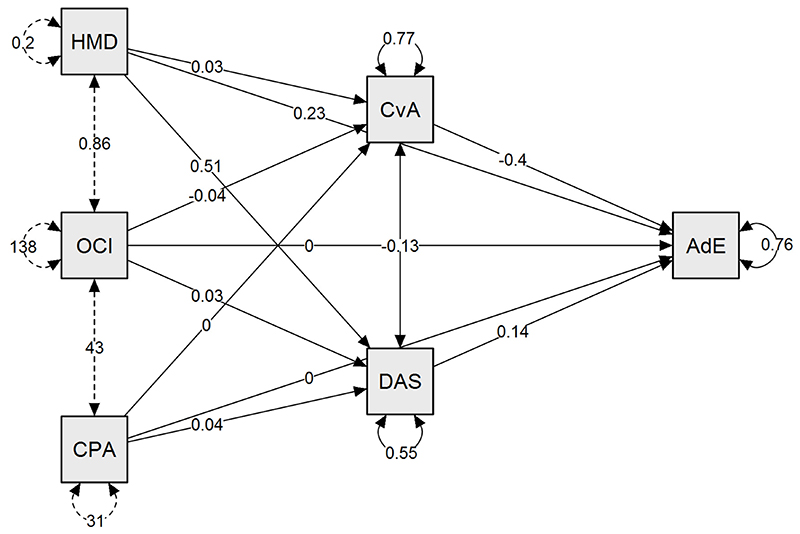
Mediation model of the extreme groups This path plot is a graphical representation of the mediation model for the extreme groups (Table 7). The model shows that the effect of history of mental disorder (HMD) on adjustment is direct, while the effect of OC symptoms (OCI-R) is indirect through fear of Covid (CAS). In the analysis adjustment is defined as a categorical outcome (answer to the first bespoke question, see methods). The arrows indicate the effects, that can be direct or indirect. The total effect is the sum of the direct and the indirect effect: mediation analysis decomposes an existing effect into these two terms. In the path plot the standardized values of the relationship (strength of the paths) are reported on the links. AdT: Adjustment CvA: Covid Anxiety Scale (fear of Covid) DAS: DASS-21 HMD: History of mental disorder CPA: CPAS OCI: OCI-R

**Table 1:** Statements describing the presence and severity of experienced adjustment difficulties

1. I am having great difficulty adjusting to the easing of the Covid-19 pandemic restrictions
2. I am finding it harder to manage my fears about COVID now that the Covid-19 pandemic restrictions are easing than I did when the restrictions were fully in force.
3. I am finding it very stressful going out of the house now that the Covid-19 pandemic restrictions are easing.
4. I am thinking too much about contracting or spreading Coronavirus now that the Covid-19 pandemic restrictions are easing.
5. I am thinking too much about other risks to my or others’ physical health now that the Covid-19 pandemic restrictions are easing.
6. I am finding it hard to stop physical distancing or avoiding contact with people now that the Covid-19 pandemic restrictions are easing.
7. I am finding it hard to stop disinfecting behaviours (e.g. handwashing, use of sterile wipes, use of gloves, masks, etc.) that are no longer officially recommended now that the Covid-19 pandemic restrictions are easing.

Participants were asked to choose one of the following 5 alternative responses for each statement: Completely disagree, disagree, neither agree nor disagree, agree, completely agree. Scores on the responses were allocated from completely disagree = 1 to completely agree = 5).

**Table 2:** Sociodemographic data.

**Gender**	**%**		
Male	28		
Female	71		
Prefer not to say	1		
**Country**	**%**	**Ethnicity**	**%**
England	87	White	85
Scotland	4	Mixed	4
Italy	6	Asian	5
India	1	Black	3
USA	1	Other	2
Rest of the World	1	Prefer not to say	1
**Highest Level of Education**	**%**	**Living Status**	**%**
GCSEs	3	Alone	15
A Level	10	With family of birth	17
Bachelor (BSc)	34	With own family	10
Master (MSc)	33	With friends	46
PhD	11	Other	11
Other	9	Prefer not to say	1
**Occupation**	**%**	**Living Status**	**%**
Employed	54	Alone	15
Unemployed	10	With family of birth	17
Furloughed	8	With own family	10
Retired	4	With friends	46
Frontline NHS	4	Other	11
NHS working with COVID patients	10	Prefer not to say	1

**Table 3:** COVID-19 related data.

**Have you or any member of your family contracted COVID-19?**	**%**
Yes	14
No	75
Unsure	11
**Has someone close to you died of COVID-19 or a COVID-related illness?**	
Yes	9
No	91
**Do you have a history of any mental health disorder?**	
Yes	30
No	64
Prefer not to say	6
**Does any member of your family have any history of mental health disorder?**	
Yes	36
No	56
Prefer not to say	8
**How well have you complied to government guidance during lockdown?**	
Extremely well	40
Very well	42
Moderately well	13
Slightly well	3
Not well at all	2

**Table 4:** Clinical ratings for the total sample (N=514)

	Mean	Standard Deviation
**DASS-21 Depression**	11.01	5.8
**DASS-21 Anxiety**	9.21	4.8
**DASS-21 Stress**	12.1	5.8
**DASS-21 Total**	32.2	15.7
		
**CPAS**	16.1	8.5
**OCI-R**	35.5	11.7
**Covid-19**	19.6	5.9

DASS-21: Depression, Anxiety and Stress Scale - 21 Items

CPAS: Compulsive Personality Assessment Scale

OCI-R: Obsessive-Compulsive Inventory - Revised

**Table 5:** Correlations between adjustment ratings and scores on the clinical scales in the total sample (N=514).

Adjustment Score (sum of the individual scores of the 7 bespoke adjustment questions)	**DASS-21**	Pearson *r* .47	p<0.001
	**OCI-R**	Pearson *r* .35	p<0.001
	**CPAS**	Pearson *r* .43	p<0.001

The Adjustment Score was calculated by obtaining the sum of the answers to the adjustment questions (from completely disagree = 1 to completely agree = 5).

DASS-21: Depression, Anxiety and Stress Scale - 21 Items

CPAS: Compulsive Personality Assessment Scale

OCI-R: Obsessive-Compulsive Inventory - Revised

**Table:** 

Direct effects								
							95% Confidence Interval	
			Estimate	Std. Error	z-value	p	Lower	Upper
CPAS tot	→	Total Adjustment	0.081	0.052	1.538	0.124	-0.022	0.183
Previous Mental Disorder	→	Total Adjustment	0.738	0.506	1.457	0.145	-0.255	1.730
OCI-R tot	→	Total Adjustment	-0.005	0.027	-0.174	0.862	-0.058	0.048

*Note*. The mediation analysis employed the Maximum Likelihood (ML) estimator and the Delta method for standard errors, as the test of the mediator’s significance. Normal theory was used to derive the 95% confidence intervals.

**Table:** 

Indirect effects										
									95% Confidence Interval	
					Estimate	Std. Error	z-value	p	Lower	Upper
**CPAS tot**	→	**DASS-21**	→	Adjust	**0.042**	**0.015**	**2.816**	**0.005**	**0.013**	**0.072**
CPAS tot	→	COVID	→	Adjust	0.004	0.040	0.091	0.927	-0.074	0.081
**Previous Mental** **Disorder**	→	**DASS-21**	→	**Adjust**	**0.512**	**0.169**	**3.029**	**0.002**	**0.181**	**0.844**
Previous Mental Disorder	→	COVID	→	Adjust	-0.114	0.375	-0.304	0.761	-0.850	0.621
**OCI-R tot**	→	**DASS-21**	→	**Adjust**	**0.035**	**0.011**	**3.224**	**0.001**	**0.014**	**0.056**
**OCI-R tot**	→	**COVID**	→	**Adjust**	**0.155**	**0.021**	**7.367**	**< .001**	**0.114**	**0.196**

*Note*. As above, the analysis employed the ML estimator, Delta method for standard errors, and normal theory for confidence intervals.

Results that are significant are depicted in bold

**Table:** 

Direct effects								
							95% Confidence Interval	
			Estimate	Std. Error	z-value	p	Lower	Upper
CPAS tot	→	Categorical Adjustment	0.010	0.006	1.778	0.075	-0.001	0.021
**Previous Mental Disorder**	→	Categorial Adjustment	**0.145**	**0.055**	**2.636**	**0.008**	**0.037**	**0.253**
OCI-R tot	→	Categorical Adjustment	-0.003	0.003	-1.192	0.233	-0.009	0.002

*Note*. As per Table 6 above, the analysis employed the ML estimator, Delta method for standard errors, and normal theory for confidence intervals.

**Table:** 

Indirect effects										
									95% Confidence Interval	
					Estimate	Std. Error	z-value	p	Lower	Upper
CPAS tot	→	DASS-21	→	Cat Adjustment	0.002	0.001	1.422	0.155	-0.897	0.005
CPAS tot	→	COVID	→	Cat Adjustment	0.005	0.002	0.091	0.927	-0.003	0.004
Previous Mental Illness	→	DASS-21	→	Cat Adjustment	0.024	0.016	1.447	0.148	-0.008	0.055
Previous Mental Disorder	→	COVID	→	Cat Adjustment	-0.005	0.018	-0.304	0.761	-0.040	0.029
OCI-R tot	→	DASS-21	→	Cat Adjustment	0.002	0.001	1.467	0.142	-0.576	0.004
**OCI-R tot**	→	**COVID**	→	**Cat Adjustment**	**0.007**	**0.001**	**5.275**	**< .001**	**0.005**	**0.010**

*Note*. As per Table 6, above, the analysis employed the ML estimator, Delta method for standard errors, and normal theory for confidence intervals.

Results that are significant are depicted in bold
